# Identification and genomic characterization of a goose astrovirus in Henan Province, China

**DOI:** 10.3389/fvets.2026.1806778

**Published:** 2026-04-15

**Authors:** Wang Dong, Huifang Lv, Xiaojie Zhang, Yamin Sheng, Qimeng Wu, Jingyi Li, Yuchen Wu, Zhifeng Peng

**Affiliations:** 1 College of Veterinary Medicine, Henan University of Animal Husbandry and Economy, Zhengzhou, China; 2 College of Veterinary Medicine, Huazhong Agricultural University, Wuhan, China

**Keywords:** full genome sequencing, goose astrovirus, isolation and identification, ORF2, phylogenetic analysis, selection pressure analysis

## Abstract

Goose astrovirus (GAstV) is an emerging pathogen that primarily causes gout in goslings under 4 weeks of age, resulting in substantial economic losses to the goose industry in China. In this study, we isolated and identified a GAstV strain, designated GAstV/HNJZ, from infected goslings in Henan Province, China. Infected goslings exhibited typical pathological lesions: the surfaces of the heart, liver, and kidneys were covered with urate membranes, and a “villous heart” was formed due to urate deposits. The kidneys and the joints were swollen. Massive urate deposits were observed in the tarsal and toe joints. Sequence analysis revealed that the complete genome of GAstV/HNJZ isolate was 7,183 nt in length, sharing the highest nucleotide identity (99.6%) with the virulent GAstV strain AAstV/Goose/CHN/2023/HR2306/1 from Anhui Province. Phylogenetic analysis demonstrated that the GAstV/HNJZ isolate belonged to genotype GAstV-II, subgenotype IId, indicating that GAstV-II remains the predominant circulating genotype in China. Several specific amino acid mutations were identified in the N-terminal capsid core domain (H26Q, A228T, A/T289T, and N380S). Selection pressure analysis revealed that Chinese GAstV strains have evolved in a relatively conserved and stable manner. This finding provides a theoretical basis for further research on the pathogenic mechanism, targeted vaccines and antiviral drugs against goose astrovirus.

## 1 Introduction

Since 2017, goslings gout disease characterized by urate deposits in internal organs and joints has occurred in goose farms in several regions of China (1). It gradually spread to 17 provinces in China including Shandong, Jiangsu, Sichuan, Anhui and so on over the past decade (2–5). Goose astrovirus (GAstV) is the pathogen responsible for gout in geese. The infection rate and mortality rate can reach 80 and 50%, respectively. Currently, there is still no commercialized vaccine or treatment strategy to prevent this disease. Strict biosecurity measures are the primary control strategy, but the continuous outbreak of GAstV still poses a huge threat to the poultry industry. GAstV is mainly pathogenic to goslings under 4 weeks of age. It has a strong resistance to the external environment, so it can persist in the environments such as water pools and excrement. The main transmission routes of GAstV are fecal-oral transmission and vertical transmission (6, 7).

GAstV, a member of the *Avastroviruses* genus within the *Astroviridae* family, is a non-enveloped, single-stranded, positive-sense RNA virus with a genome length ranging from 6.8 to 7.9 kb. The genome structure includes the 5′-untranslated region (5’-UTR), three open reading frames (ORF1a, 1b, and 2), 3’-UTR and a poly(A) tail. Among them, ORF1a and ORF1b are responsible for encoding the non-structural proteins of the virus (8), ORF2 encodes the capsid protein, which can encapsulate the nucleic acid of GAstV and invade cells through interactions with the host, triggering host immune response (9). Previous studies have shown that nanobodies or antiviral peptides ligands targeting ORF2 possess antiviral effect on GAstV infection (10, 11). Meanwhile, it is the most prone to mutation, so ORF2 is commonly used as the basis for GAstV typing. GAstV has two genotypes (GAstV-I and GAstV-II). Among them, GAstV-II is more virulent and is the currently prevailing dominant genotype (12). GAstV-I type viruses are rarely found in single infections, and there is also a mixed infection of these two types of GAstV, which can cause more typical gout symptoms (13).

In this study, a GAstV strain was isolated from the tissue samples of a goose farm in Jiaozuo, Henan Province, and named GAstV/HNJZ. The whole genome sequence was amplified and analyzed, and genetic evolution was analyzed at the whole genome nucleotide and ORF2-encoded amino acids, respectively, to understand the epidemic characteristics and variation features of the prevalent GAstV strains. This study is of great significance for the prevention and control of GAstV in China.

## 2 Materials and methods

### 2.1 Case history and samples

In June 2023, a fatal gout disease of goslings occurred in a commercial goose farm with 10,000 geese in Jiaozuo, Henan Province. The geese were raised in well-ventilated houses, provided with formula feed and ad libitum access to water. Strict routine biosafety measures, including disinfection and restrictions on personnel entry and exit, had been carried out. However, 40% of the geese exhibited anorexia, reluctance to move, and even paralysis accompanied by significantly swollen leg joints, along with white lime-like or yellowish-green watery diarrhea. The moribund goslings were randomly selected and sent to the laboratory for diagnosis.

### 2.2 Virus detection

To identify the causative agent of the disease, potential pathogens were screened from supernatants of homogenized tissue samples. Viral DNA/RNA was extracted using a viral DNA/RNA extraction kit (Takara, Dalian, China) and reverse transcribed into cDNA. Subsequently, PCR detection was performed for goose parvovirus (GPV), goose reovirus (GRV), avian paramyxovirus-1 (APMV-1), Tembusu virus (TMUV), goose calicivirus (GCV), goose hemorrhagic polyomavirus (GHPV), fowl adenovirus (FAdV), and goose astrovirus (GAstV) using specific primers (Table 1).

**Table 1:** Primers used in this study for detection of the potential viral pathogen.

Primers	Sequences (5′-3′)	Amplicon size (bp)
GPV-F	AGACTTATCAACAACCATT	779
GPV-R	TCACTTATTCCTGCTGTAG	
GRV-F	TGAGACGCCTGACTACGATT	380
GRV-R	ATGCTTGGAGTGAGACGACT	
APMV-1-F	CACCGGCAACCCTATTCTGT	330
APMV-1-R	AGTGCGCCTTCAGTCTTTGA	
TMUV-F	GCCACGGAATTAGCGGTTGT	401
TMUV-R	TAATCCTCCATCTCAGCGGTGTAG	
GCV-F	TGCATCTGGGACGAATTTGA	134
GCV-R	ACGCTGGAGGTGAACATT	
GHPV-F	GAGGTTGTTGGAGTGACCACAATG	144
GHPV-R	ACAACCCTGCAATTCCAAGGGTTC	
FAdV-F	GCAGCGTGGTCTTGAAGATGGTTC	632
FAdV-R	CGCATTCAAGCCCGTTCGATTC	
GAstV-F	ACGACAGATGCGTTACTT	277
GAstV-R	GGTGACATTATCCCTGAG	

### 2.3 Virus isolation

The GAstV-positive tissue samples were homogenized aseptically in phosphate-buffered saline (PBS, pH 7.2), freeze-thawed three times, and centrifuged at 8,000 × g for 10 min. The supernatants were filtered through a 0.22 μm filter (Millipore, Massachusetts, United States). Then, 0.2 mL supernatant per egg was inoculated into the allantoic cavity of 15-day-old healthy goose embryos, which were negative for GAstV, GPV, GRV, APMV-1, TMUV, GCV, GHPV, and FAdV. Allantoic fluids were collected aseptically from embryos that died 5–7 days post-inoculation for next generation inoculation. After three generations, the embryos were collected for the observation of lesions, and the allantoic fluids were harvested sterilely to extract viral DNA/RNA for identifying the potential pathogens, including GPV, GRV, APMV-1, TMUV, GCV, GHPV, FAdV, and GAstV.

### 2.4 Complete genome sequencing

To determine the complete genome of the newly isolated GAstV strain, total RNA was extracted from clinical samples as described above and reverse transcribed into cDNA using ReverTra Ace® qPCR RT Master Mix with gDNA Remover (TOYOBO, Osaka, Japan) with random primers according to the manufacturer’s instructions. Viral genomic fragments were amplified by overlap PCR using six pairs of specific primers designed based on the conserved regions of reference GAstV strains available in the GenBank database (Supplementary Table S1). The 5’-UTR and 3’-UTR regions were amplified using a 5’RACE and 3’RACE kit (Invitrogen, California, United States) following the manufacturer’s protocol. The PCR amplicons were purified and ligated into pMD18-T vector (Takara, Dalian, China) for subsequent sequencing.

### 2.5 Sequence comparison and phylogenetic analysis

The whole genome sequence of the GAstV isolate was aligned against the NCBI database using BLAST.^1^ A total of 14 classic reference GAstV strains (3 GAstV-I and 11 GAstV-II) were downloaded from GenBank as reference strains for subsequent analysis. The homology of the whole genome nucleotide and the amino acid sequence encoded by ORF2 were compared between the GAstV isolate and the reference strains using DNAStar-MegAlign. Phylogenetic trees were constructed via the maximum likelihood (ML) method in MEGA 12.0 with 1,000 bootstrap replicates: the GTR + I model was used for the whole genome nucleotide sequence, and the JTT model for the ORF2-encoded amino acid sequence. Additionally, amino acid mutations in the GAstV ORF2 protein were analyzed.

### 2.6 Selection pressure analysis

Selection pressure analysis was conducted using the BUSTED (branch-site unrestricted statistical test for episodic diversification) and FUBAR (fast unconstrained Bayesian approximation) methods on the Datamonkey online server^2^ to determine whether the GAstV ORF2 gene is under positive selection pressure and identify potential positive selection sites. The BUSTED method estimates the *ω* value, which is the ratio of nonsynonymous substitutions (dN) to synonymous substitutions (dS). When ω > 1, positive selection is indicated; when ω = 1, neutral selection is indicated; and when ω < 1, negative (purifying) selection is indicated. The FUBAR method designates sites as under positive selection with a posterior probability ≥0.9. To minimize the risk of false positive results, only sites identified as positively selected by both BUSTED and FUBAR were considered reliable.

## 3 Results

### 3.1 Clinical signs and post-mortem examinations

**Figure 1:**
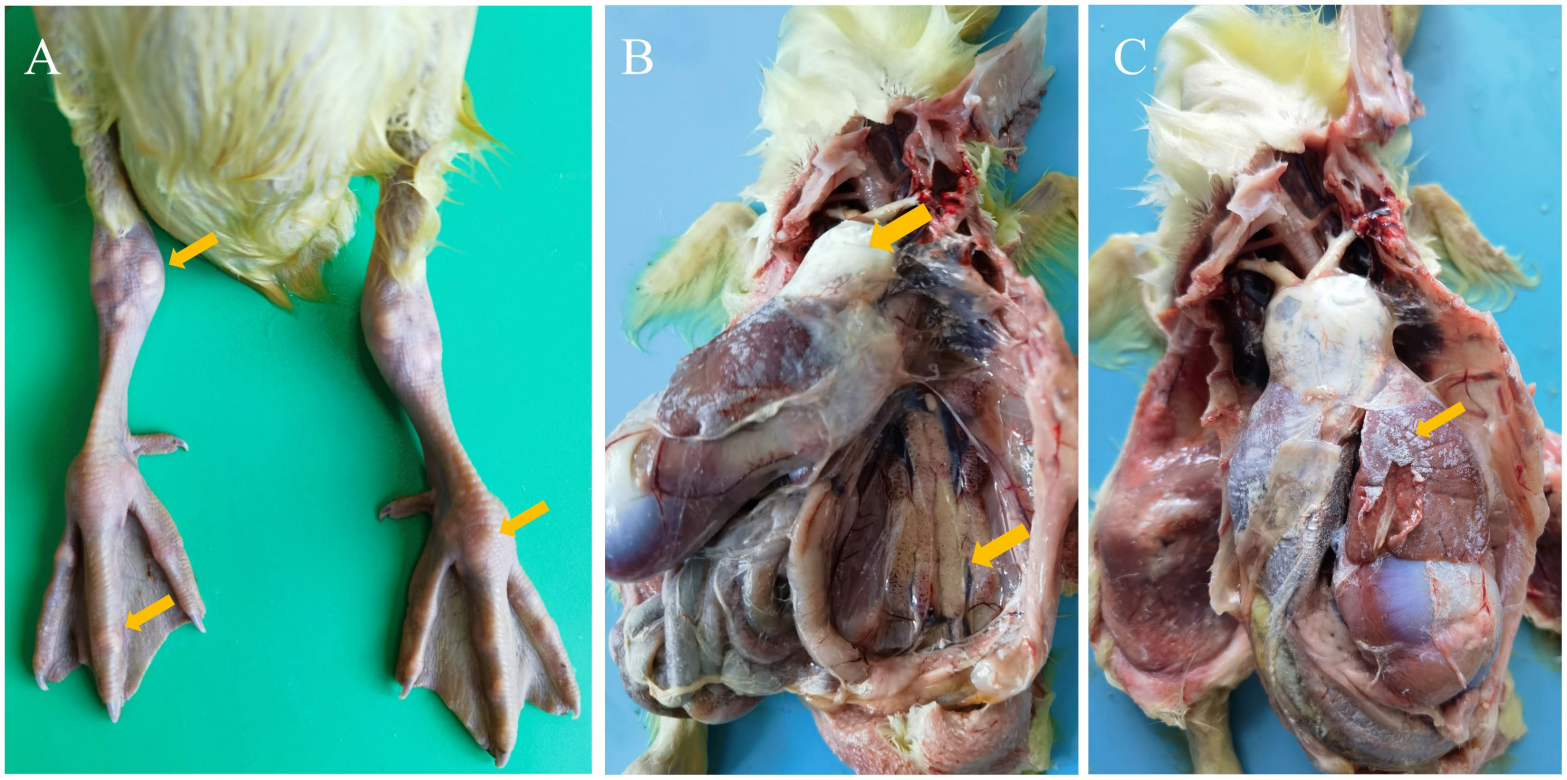
Gross lesions of clinical samples. **(A)** Joint swelling and urate deposits in the joints (yellow arrows). **(B,C)** Urate deposits on the heart, kidneys, and liver, along with kidneys swelling. Yellow arrows indicate urate deposits in the heart and kidneys, and kidneys swelling **(B)**, as well as urate deposits in the liver **(C)**.

For this disease outbreak, most sick goslings exhibited typical clinical signs, including lime-like feces, depression, anorexia, ruffled and loose feathers, unsteady gait, or even paralysis. Their joints were swollen, with a large amount of urate deposits in the joint cavities (Figure 1A). Post-mortem examination revealed typical visceral and articular gout. The surfaces of the heart and liver of the sick goslings were covered with urate membranes, and a “villous heart” formed due to urate deposits (Figures 1B,C). The kidneys were swollen, and urate deposits were observed (Figure 1B).

### 3.2 Pathogen detection and virus isolation

**Figure 2:**
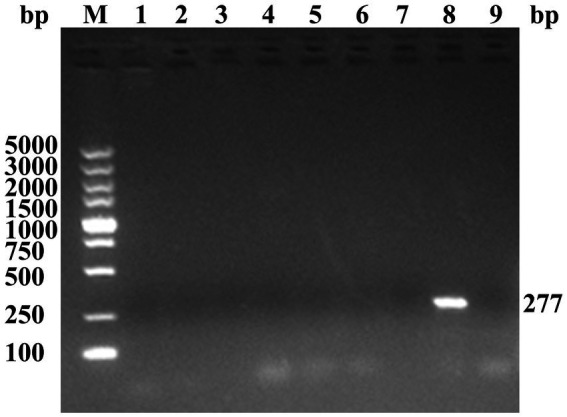
PCR/RT-PCR detection of the potential pathogen. M, DL5000 DNA marker; lane 1, GPV; lane 2, GRV; lane 3, APMV-1; lane 4, TMUV; lane 5, GCV; lane 6, GHPV; lane 7, FAdV; lane 8, GAstV; lane 9, negative control. Purified DNA/RNA samples were used to detect the potential pathogen causing gout in goslings using PCR/RT-PCR methods with specific primers, including GPV (779 bp), GRV (380 bp), APMV-1 (330 bp), TMUV (401 bp), GCV (134 bp), GHPV (144 bp), FAdV (632 bp), and GAstV (277 bp).

To identify the causative pathogen of the disease, PCR/RT-PCR assays were performed to screen for potential pathogens. Tissue samples from diseased goslings tested positive for GAstV but negative for GPV, GRV, APMV-1, TMUV, GCV, GHPV, and FAdV (Figure 2). All tested goslings (5/5, 100%) were positive for GAstV by RT-PCR, indicating that this disease was caused by GAstV infection.

**Figure 3:**
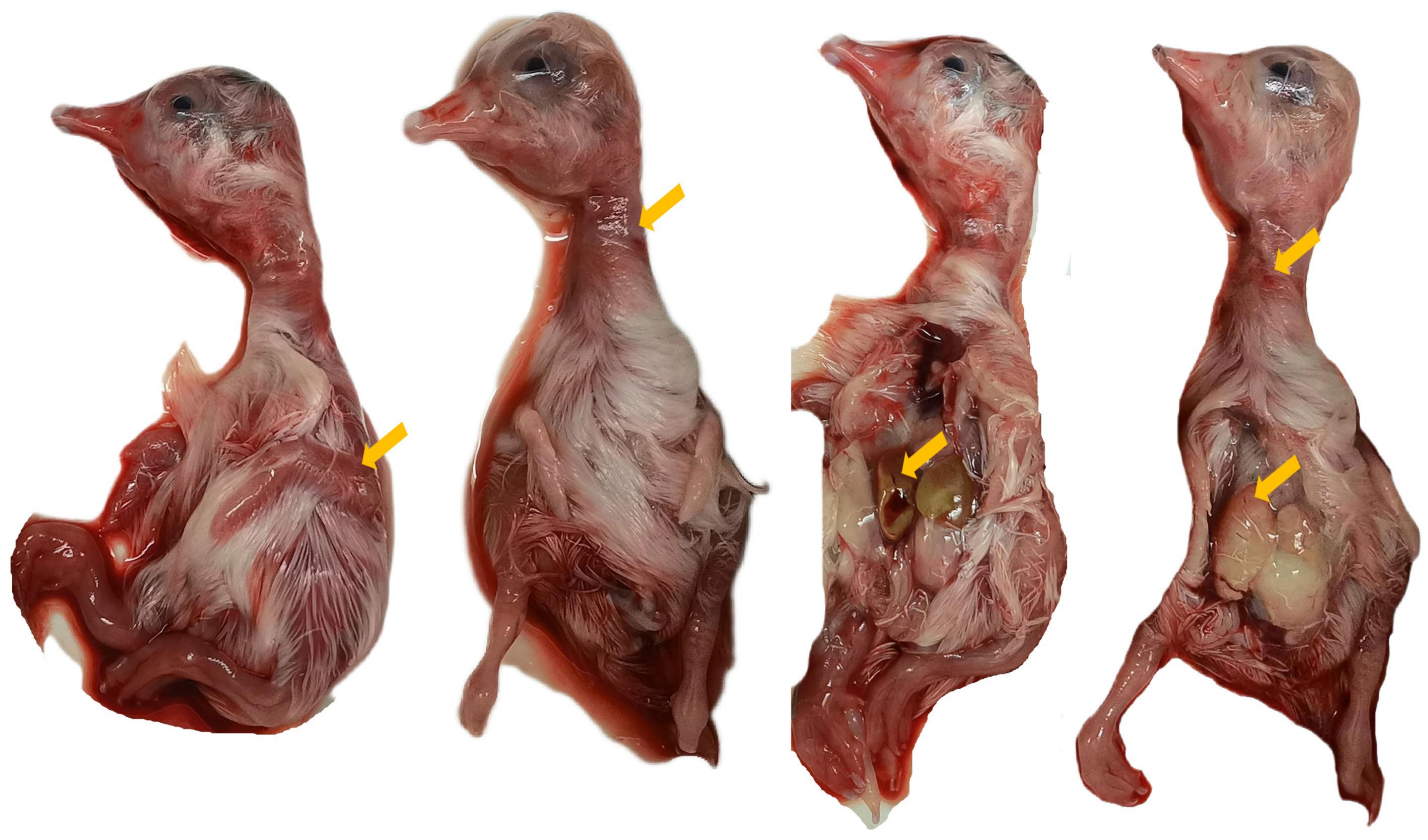
Pathogenicity of GAstV in goose embryos. Goose embryos inoculated with fourth-passage GAstV strain all died within 5–7 days, showing embryonic hemorrhage, hepatic edema, and hemorrhage (yellow arrows).

Homogenates prepared from GAstV-positive tissue samples were inoculated into goose embryos. After three blind passages, all inoculated embryos died within 5–7 days, presenting with embryonic hemorrhage, hepatic edema and hemorrhage (Figure 3). The allantoic fluids collected from these embryos tested positive for GAstV, confirming successful isolation of a GAstV strain, which was designated as the GAstV/HNJZ isolate.

### 3.3 Complete genome sequencing and characterization

The complete genomic sequence of the GAstV/HNJZ isolate was amplified and sequenced using the overlapping PCR and RACE strategies. Sequence analysis revealed that the full-length genome of the GAstV/HNJZ isolate was 7,183 nt in length. This genome contained three ORFs, among which ORF1a was 3,255 nt in length (19–3,273 nt), ORF1b was 1,551 nt (3,264–4,814 nt), and ORF2 was 2,115 nt (4,833–6,947 nt). The UTRs were located at the 5′ and 3′ ends of the genomic sequence with a 5’-UTR of 18 nt (1–18 nt) and a 3’-UTR of 236 nt (6948–7,183 nt).

**Figure 4:**
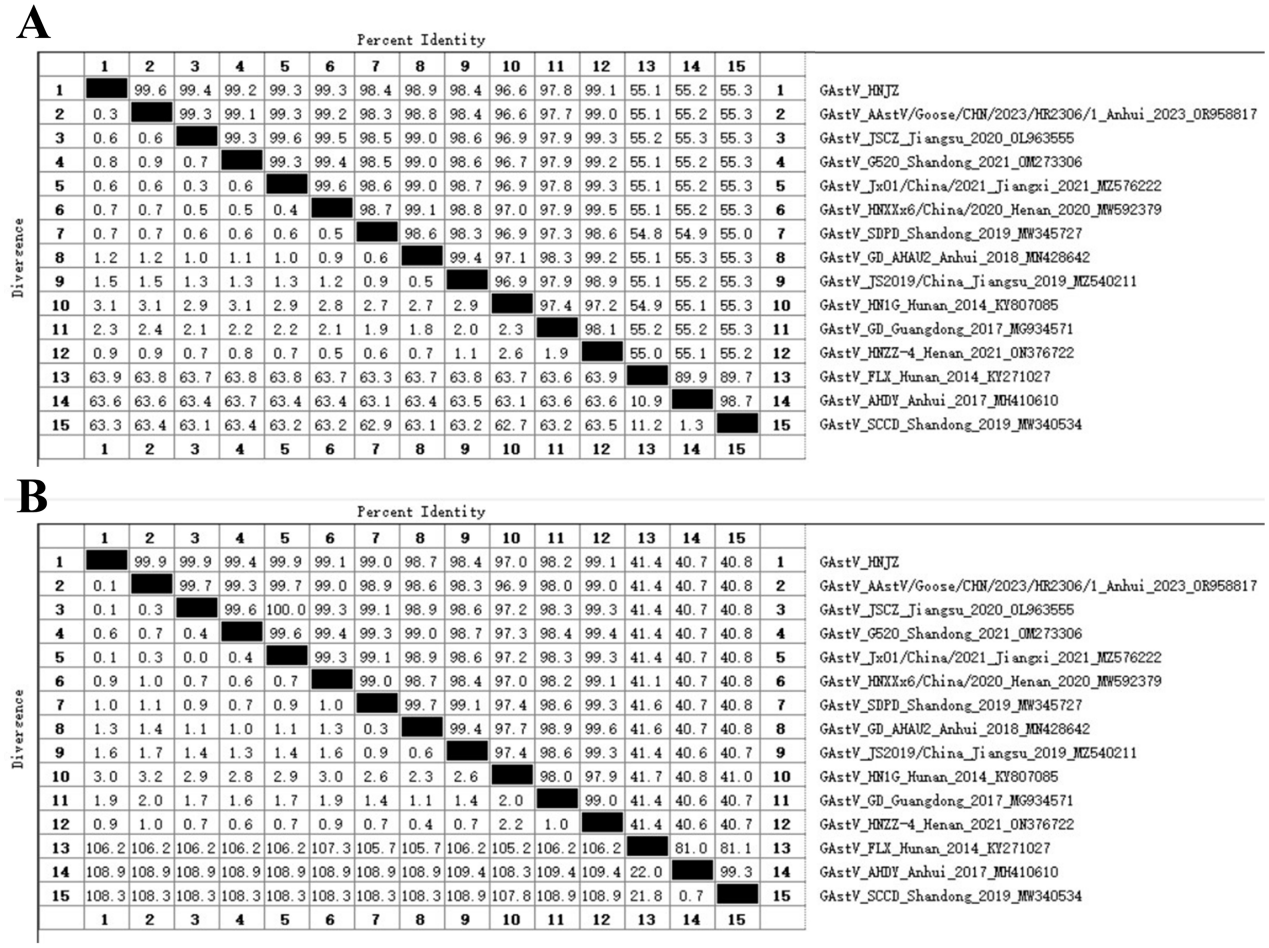
Homology analysis between the GAstV/HNJZ isolate and reference strains. Sequence similarity analysis of the whole genome nucleotide sequence **(A)** and ORF2-encoded amino acid sequence **(B)** between the GAstV/HNJZ isolate and 14 reference GAstV strains from GenBank were performed using MegAlign in DNAStar.

The homology of the GAstV/HNJZ isolate was compared with other representative GAstV strains using the NCBI BLAST tool. The GAstV/HNJZ isolate shared the highest nucleotide identity (99.6%) with isolate AAstV/Goose/CHN/2023/HR2306/1 from Anhui Province. Whole genome nucleotide homology and ORF2 amino acid homology between the GAstV/HNJZ isolate and 14 reference strains were analyzed using DNAStar-MegAlign. The results showed that the whole genome homology between the GAstV/HNJZ isolate and 11 GAstV-II strains ranged from 96.6 to 99.6% (Figure 4A), whereas homology with the three classic GAstV-I representative strains (FLX, AHDY, SCCD) was very low, at only 55.1 to 55.3%, indicating that the GAstV/HNJZ isolate belongs to the GAstV-II genotype. In addition, ORF2 amino acid homology between the GAstV/HNJZ isolate and GAstV-II reference strains ranged from 97.0 to 99.9% (Figure 4B). The highest homology (99.9%) was observed with isolates AAstV/Goose/CHN/2023/HR2306/1, JSCZ, and JX01/China/2021, while the lowest was with isolate HN1G (97.0%). Homology with GAstV-I strains remained very low, ranging from 40.7 to 41.4%.

### 3.4 Phylogenetic analysis

**Figure 5:**
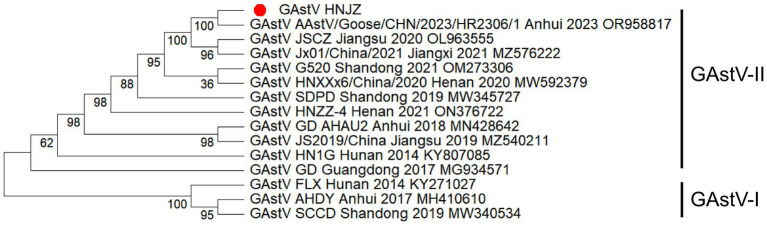
Phylogenetic analysis of the whole genome sequences between the GAstV/HNJZ isolate and GAstV reference strains. Based on the multiple alignment of the whole genome sequence of GAstV/HNJZ and 14 reference GAstVs available from the GenBank database, a phylogenetic tree was constructed using MEGA 12.0 software via the maximum likelihood method with 1,000 bootstrap replicates. Two distinct lineages (GAstV-I and GAstV-II) are indicated. The new isolate in this study is indicated by a red solid circle.

**Figure 6:**
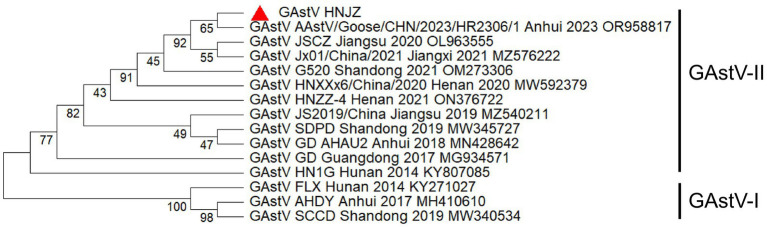
Phylogenetic analysis of GAstVs based on ORF2-encoded amino acid sequences. A phylogenetic tree was constructed using MEGA 12.0 software via the maximum likelihood method with 1,000 bootstrap replicates. The new isolate in this study is indicated by a red solid triangle.

To further explore the evolutionary characteristics of the newly identified GAstV/HNJZ isolate, a phylogenetic tree based on the complete genome sequence was constructed. As shown in Figure 5, GAstVs were divided into two distinct lineages: GAstV-I represented by FLX, AHDY, SCCD, and GAstV-II type represented by HN1G, GD, SDPD, and others. The GAstV/HNJZ isolate clustered within the GAstV-II lineage and was grouped closely with AAstV/Goose/CHN/2023/HR2306/1 in a well-supported subclade (bootstrap value = 100), indicating a close evolutionary relationship. This subclade further clustered with other recent Chinese strains isolated from Jiangsu (JSCZ, 2020), Jiangxi (Jx01, 2021), and Shandong (G520, 2021) within a larger clade (bootstrap value = 95), indicating that these strains represent the dominant circulating genotype of GAstV in China from 2019 to 2023. In contrast, earlier strains from Hunan (FLX, 2014), Anhui (AHDY, 2017), and Shandong (SCCD, 2019) formed a distinct basal clade (bootstrap value = 100), suggesting that GAstV has undergone temporal evolutionary divergence in China. However, the GAstV/HNJZ isolate belonged to a different subclade from the HN1G and GD strains, with lower genetic relatedness than that observed between AAstV/Goose/CHN/2023/HR2306/1 and Jx01/China/2021. Notably, GAstV-II is further divided into subgenotypes IIa, IIb, IIc and IId. Strain HN1G belongs to subgenotype IIa, strain GD to IIb, and the GAstV/HNJZ isolate together with most other Chinese strains to IId. The phylogenetic tree based on the ORF2-encoded amino acid sequence (Figure 6) exhibited a similar topological structure to that of the whole genome tree, further supporting the key role of ORF2 in GAstV genotyping and classification.

### 3.5 Amino acid polymorphism analysis

To better understand the amino acid polymorphism of the newly identified GAstV/HNJZ isolate, the ORF2-encoded amino acid sequence was compared with those of 11 other GAstV-II strains. The results showed that the isolate harbored four amino acid mutations in the N-terminal capsid core domain of the capsid protein compared with the 11 GAstV-II strains mentioned above: H26Q, A228T, A/T289T, and N380S (Table 2). The mutations at position A228T may be related to antigenic epitope alteration (14). The mutation at position A/T289T of ORF2 can act as a marker for genetic evolution (15). In addition, since the HN1G strain belongs to subgenotype IIa, the GD strain to IIb, and the remaining strains to IId, three amino acid mutations (E456D, A464N and L540Q) were identified between the GAstV/HNJZ isolate and HN1G or GD strain. These three mutation sites were components of epitopes, in the highly variable C-terminal capsid spike domain, indicating that these mutations might alter viral antigenicity (16).

**Table 2:** ORF2 amino acid mutations between GAstV/HNJZ and 11 reference GAstV-II strains.

Position	GAstV-IId		GAstV-IIa	GAstV-IIb	Location
	HNJZ	others	HN1G	GD	
26	Q	H/Q	H	H	Cap_C
228	T	A/T	A	A	Cap-C
289	T	A/T	A	A	Cap_C
380	S	N/S	N	N	Cap_C
456	D	D	E	E	Cap_P2
464	N	N	A	A	Cap_P2
540	Q	Q	L	L	Cap_P2

Cap_C represents the N-terminal capsid core domain, Cap_P2 represents the C-terminal capsid spike domain of the capsid protein.

### 3.6 Selection pressure analysis

Selection pressure analysis using the BUSTED method revealed that the ORF2 gene was under strong purifying selection overall, with a mean *ω* value of 0.1983 (<1). Specifically, 80.85% of sites exhibited extremely strong purifying selection (ω < 0.01), while only 19.15% of sites were under neutral selection (ω = 1), indicating high functional conservation of the ORF2 gene and strict purging of nonsynonymous mutations to maintain protein functional stability. The mean synonymous substitution rate was 1.000, with a coefficient of variation (CoV) of 0.3286, suggesting reliable model estimation and a relatively uniform background evolutionary rate. Notably, no positive selection sites were identified by both the BUSTED and FUBAR methods, indicating that no positively selected sites exist in the ORF2 gene of the GAstV strains analyzed in this study.

## 4 Discussion

Since the large-scale outbreak of goose astrovirus (GAstV) infection in domestic goslings in 2017, preventing viral infection has become one of the urgent issues in goose breeding industry. Affected goslings exhibit signs of depression, inactivity, and reduced appetite, which seriously impair production efficiency. Post-mortem examination reveals that the abdominal cavity of diseased goslings is covered with “frost-like” white or pale yellow urate deposits, especially on the surface of internal organs such as the heart, liver, spleen, and kidneys; urate deposits are also present in the joint cavities, accompanied by kidneys and joints enlargement (17). In this study, the sick goslings displayed typical clinical and post-mortem symptoms, laying a foundation for the further identification of GAstV.

The positive rate of GAstV is relatively higher in autumn and winter than in other seasons due to poor ventilation caused by indoor insulation or cold climate conditions, which is in accordance with the epidemic characteristics of other viral diseases such as influenza (13). GAstV also poses a risk of cross-species transmission. GAstV can cause similar gout symptoms in laying hens, Muscovy ducks and cherry valley ducks besides geese (18–21). This undoubtedly facilitates virus mutation and recombination, posing a potential threat to the poultry industry in China. Meanwhile, it is necessary to closely monitor the prevalence of new variant strains. In this study, a GAstV strain was successfully isolated from 15-day-old diseased goslings with visceral and joint gout in Henan Province, designated GAstV/HNJZ.

In this study, the whole genome sequence of the GAstV/HNJZ isolate was 7,183 nt at the full length, with a typical GAstV gene structure, namely 5’-UTR-ORF1a-ORF1b-ORF2-3’-UTR. Nucleotide similarity comparison revealed that it ranged from 96.6 to 99.6% between the GAstV/HNJZ isolate and the currently prevalent GAstV-II strain in China. This high similarity helps to better understand the evolution and transmission mode of the virus. In order to further study the evolutionary history of the GAstV/HNJZ isolate, this experiment selected representative isolates from Shandong, Jiangsu, Anhui, Henan, Hunan, Guangdong and other places as references to conduct phylogenetic tree. Phylogenetic analysis of the whole genome of the GAstV/HNJZ isolate with the reference sequence revealed that the GAstV/HNJZ isolate belonged to genotype GAstV-II, subgenotype IId, possessing close evolutionary relationships with AAstV/Goose/CHN/2023/HR2306/1 and a significantly different evolutionary relationship with GAstV-I type. It indicated that the two groups of GAstV derived from different evolution histories and that there has been no significant variation of GAstV in the geese in Henan Provice at present, with that the predominant strain is still GAstV-II. Phylogenetic analysis also revealed that GAstV had experienced temporal evolutionary differentiation in China. GAstV-I was popular in the early period, while GAstV-II became popular later. Strains from different provinces were widely distributed in each branch, indicating frequent cross-regional transmission of GAstV in China. Due to the high pathogenicity and wide distribution of GAstV in goslings (22), close attention to this virus is still needed. Characterizing the evolution of GAstV is beneficial for further understanding this emerging pathogen.

GAstV ORF2 encodes the viral capsid protein, which mediates the binding to the host cell membrane and is the main virulence protein of GAstV. As the ORF2 gene is the most prone to variation, the ORF2 gene is usually used to determine the typing of astroviruses internationally, and the genetic evolution analysis of GAstV is also based on the ORF2 gene (23). The single ORF2 amino acid phylogenetic tree constructed in this study also classified GAstV/HNJZ isolate into GAstV-II type, which has a relatively distant evolutionary relationship with GAstV-I type. Mutations in the ORF2 can alter the ability of virus to attach to and enter host cells. In this study, the GAstV/HNJZ isolate has a close similarity (more than 97%) to the GAstV-II reference strain based on the homology of ORF2-encoded amino acids. It can be regarded as the same variant strain, also suggesting that the regional differences in the prevalence of this strain are very small, with almost no differences across the country. This study found that there were four amino acid mutations in the N-terminal capsid core domain of the capsid protein, which were H26Q, A228T, A/T289T, and N380S. The mutation at position A228T may be related to antigenic epitope alteration (14), while mutation at position A/T289T of ORF2 can act as a marker for genetic evolution (15). Whether these amino acid mutations affect the pathogenicity of the virus and host interactions needs further verification.

In addition, recombination analysis was performed in this study using RDP4 software. However, no potential recombination events were identified with GAstV/HNJZ isolate as a recombinant, indicating that no recombination may have occurred within the GAstV/HNJZ isolate. Selection pressure analysis results showed that GAstV ORF2 gene was in a strong purifying selection rather than adaptive positive selection, and the vast majority of sites were functionally conserved. Although multiple amino acid mutations were observed in GAstV/HNJZ ORF2, the overall low *ω* value (mean = 0.1983) indicated that the vast majority of nonsynonymous mutations were eliminated by natural selection due to their deleterious effects, and only a small number of neutral mutations were randomly fixed in the population, resulting in the observed sequence divergence. These analyses suggested that Chinese GAstV strains have evolved relatively conserved and stable.

Current research has focused on the molecular epidemiology, pathogenic mechanism, host immune response, and vaccine development of GAstV (24–27). Conducting etiological identification on GAstV is of great significance for its prevention and control as well as vaccine development. In the present study, a GAstV strain, designated GAstV/HNJZ, was isolated from a goose farm in Jiaozuo, and its genotype and molecular characteristics were analyzed. Although embryo lethality was observed, quantitative analysis of viral load and histopathological characterization were not performed in this study. Further comprehensive pathogenicity assessments are thus warranted to fully clarify the pathogenic potential of this virus. Nevertheless, this study provides the foundation for further exploration of the pathogenic mechanism and control strategies.

## Data Availability

The datasets presented in this study can be found in online repositories. The names of the repository/repositories and accession number(s) can be found in the article/Supplementary material.

## Footnotes

1
https://blast.ncbi.nlm.nih.gov/Blast.cgi

2
http://www.datamonkey.org

## References

[1] Liu C Sun M Liao M. A review of emerging goose astrovirus causing gout. Biomed Res Int. (2022) 2022:1635373. doi: 10.1155/2022/1635373, 36072471 PMC9441354

[2] Niu X Tian J Yang J Jiang X Wang H Chen H . Novel goose astrovirus associated gout in gosling, China. Vet Microbiol. (2018) 220:53–6. doi: 10.1016/j.vetmic.2018.05.006, 29885801

[3] Wang Y Bai C Zhang D Yang K Yu Z Jiang S . Genomic and phylogenetic characteristics of a novel goose astrovirus in Anhui Province, central-eastern China. Gene. (2020) 756:144898. doi: 10.1016/j.gene.2020.144898, 32569721

[4] Ren D Zhang H Ye X Jia X Chen R Tang T . Current situation of goose astrovirus in China: a review. Viruses. (2025) 17:84. doi: 10.3390/v17010084, 39861873 PMC11768540

[5] Chen G Yin L Zhang H. Isolation and characterization of goose astrovirus genotype 1 causing enteritis in goslings from Sichuan Province, China. BMC Vet Res. (2025) 21:259. doi: 10.1186/s12917-025-04482-9, 40205381 PMC11983725

[6] Zhang X Deng T Song Y Liu J Jiang Z Peng Z . Identification and genomic characterization of emerging goose astrovirus in Central China, 2020. Transbound Emerg Dis. (2022) 69:1046–55. doi: 10.1111/tbed.14060, 33687791

[7] Cortez V Margolis E Schultz-Cherry S. Astrovirus and the microbiome. Curr Opin Virol. (2019) 37:10–5. doi: 10.1016/j.coviro.2019.05.002, 31163291 PMC6768711

[8] Wang A Wu Z Zhou Q Zhang X Zhu Y Xie J . Isolation, identification, and pathogenicity of a goose astrovirus 1 strain from goslings in Jiangsu province, China. Microb Pathog. (2025) 200:107324. doi: 10.1016/j.micpath.2025.107324, 39864762

[9] Wang A Wu Z Zhou Q Zhang X Liu L Zhu S. Development of monoclonal antibodies against goose astrovirus 2 ORF2 protein and establishment of an indirect competitive ELISA detection method. J Virol Methods. (2026) 340:115306. doi: 10.1016/j.jviromet.2025.115306, 41271110

[10] Ge Y Yang J Liu X An F Cui Z Zeng Q. Single-domain antibody fused with chicken IgG fc-fragment protects goslings against novel goose astrovirus (nGAstV). Pak Vet J. (2025) 45:257–67. doi: 10.29261/pakvetj/2025.120

[11] Wang Y Li W Li P Chen L Jin Q. Identification of antiviral peptide ligands targeting the capsid spike domain of goose astrovirus. Pak Vet J. (2025) 45:1253–61. doi: 10.29261/pakvetj/2025.247

[12] Li M Wang M Wang J Li H Guan R Yan G . Evolutionary and genotypic analyses of goose astrovirus. Poult Sci. (2025) 104:105511. doi: 10.1016/j.psj.2025.105511, 40639001 PMC12275101

[13] Xiang Y Chen M Sun M Dong J Zhang J Huang Y . Isolation, identification, and epidemiological characteristics of goose astrovirus causing acute gout in Guangdong Province, China. Poult Sci. (2024) 103:104143. doi: 10.1016/j.psj.2024.104143, 39128392 PMC11367137

[14] Xu M Wu S Ma S Shao H Qian K Wan Z . Genetic characterization of goose astrovirus strain JSSQ isolated from Jiangsu province. Microbiol China. (2022) 49:2174–82. doi: 10.13344/j.microbiol.china.210910

[15] Fei Z Jiao A Xu M Wu J Wang Y Yu J . Genetic diversity and evolution of goose astrovirus in the east of China. Transbound Emerg Dis. (2022) 69:e2059–72. doi: 10.1111/tbed.14542, 35384346

[16] Peng Z Gao D Song X Huang H Zhang X Jiang Z . Isolation and genomic characterization of one novel goose astrovirus causing acute gosling gout in China. Sci Rep. (2023) 13:10565. doi: 10.1038/s41598-023-37784-9, 37386083 PMC10310827

[17] Wei F Yang J He D Diao Y Tang Y. Evidence of vertical transmission of novel astrovirus virus in goose. Vet Microbiol. (2020) 244:108657. doi: 10.1016/j.vetmic.2020.108657, 32402337

[18] Wei F Jiang X He D Wang Q Diao Y Tang Y. The isolation and characterization of goose astrovirus genotype 2 from laying hens with nephritis in Shandong Province, China. Transbound Emerg Dis. (2023) 2023:8515116. doi: 10.1155/2023/8515116 40303698 PMC12016900

[19] Chen Q Yu Z Xu X Ji J Yao L Kan Y . First report of a novel goose astrovirus outbreak in Muscovy ducklings in China. Poult Sci. (2021) 100:101407. doi: 10.1016/j.psj.2021.101407, 34438326 PMC8383103

[20] Chen H Zhang B Yan M Diao Y Tang Y. First report of a novel goose astrovirus outbreak in Cherry Valley ducklings in China. Transbound Emerg Dis. (2020) 67:1019–24. doi: 10.1111/tbed.13418, 31705830

[21] Wei F Yang J Wang Y Chen H Diao Y Tang Y. Isolation and characterization of a duck-origin goose astrovirus in China. Emerg Microbes Infect. (2020) 9:1046–54. doi: 10.1080/22221751.2020.1765704, 32486971 PMC7448921

[22] Yang J Tian J Tang Y Diao Y. Isolation and genomic characterization of gosling gout caused by a novel goose astrovirus. Transbound Emerg Dis. (2018) 65:1689–96. doi: 10.1111/tbed.12928, 29920970

[23] Liu C Li L Dong J Jin J Xiang Y Zhang J . Isolation, characterization, and comparative analysis of two subtypes of goose astrovirus in Guangdong Province, China. Microorganisms. (2025) 13:1037. doi: 10.3390/microorganisms13051037, 40431208 PMC12114045

[24] Zhang M Zhang L Yang J Zhao D Han K Huang X . An IgY effectively prevents goslings from virulent GAstV infection. Vaccines (Basel). (2022) 10:2090. doi: 10.3390/vaccines10122090, 36560500 PMC9781778

[25] Ren D Zhang X Zhang W Lian M Meng X Li T . A peptide-based ELISA for detection of antibodies against novel goose astrovirus type 1. J Virol Methods. (2023) 312:114646. doi: 10.1016/j.jviromet.2022.114646, 36356679

[26] Li W Xu L Tan H Wang T Wu Z He Y . Identification and characterization of linear epitopes of monoclonal antibodies against the capsid protein of goose astrovirus genotype 2. Poult Sci. (2025) 104:105357. doi: 10.1016/j.psj.2025.105357, 40466262 PMC12167802

[27] Li X Yang W Zhu M Xu H Yang J Yi Z . Goose astrovirus type 2 causes intestinal injury and disrupts homeostasis in goslings. Vet Sci. (2025) 13:15. doi: 10.3390/vetsci13010015, 41600671 PMC12846611

