# Supplementary material for: Identification and genomic characterization of a goose astrovirus in Henan Province, China
Source: Front Vet Sci. 2026 Apr 15;13:1806778. doi: 10.3389/fvets.2026.1806778 (PMC13124617; doi:10.3389/fvets.2026.1806778)
Supplement: Supplementary file 1 [file Table_1.DOCX]

Table S1 Primers used in this study for testing the full-genome of GAstV

| **number** | **Sequences (5’-3’)** | **position** | **Amplicon size (bp)** |
| --- | --- | --- | --- |
| 1 | F1:GCATGGGGAAACAGCGATA | 1-1561 | 1561 |
|  | R1:TCGACACAGTACCGTCAGG |  |  |
| 2 | F2:GCGTGTTCCACTATTACAGTC | 1516-3086 | 1571 |
|  | R2:CTCGACCCACACCACTTACA |  |  |
| 3 | F3:TCAACCTCTTGAGCAGCGCA | 3039-3944 | 906 |
|  | R3:CTTCATTTTCTCATTCTGGTC |  |  |
| 4 | F4:CCCCTCTGGTGGTGTTTTCTC | 3801-4762 | 962 |
|  | R4:TCACTGGCAGCGTGCTCCT |  |  |
| 5 | F5:ATCAACACGGTTGAGGAGC | 4731-6294 | 1564 |
|  | R5:CCCACCAATTGTGTGTTCC |  |  |
| 6 | F6:CGTGGAAGGACTTCAACATC | 6246-7116 | 871 |
|  | R6:TAAACAAAAACCCGGTCAGG |  |  |
